# Changes in the role of Pacific decadal oscillation on sea ice extent variability across the mid-1990s

**DOI:** 10.1038/s41598-020-74260-0

**Published:** 2020-10-16

**Authors:** Hyerim Kim, Sang-Wook Yeh, Soon-Il An, Se-Yong Song

**Affiliations:** 1 ERICA, Hanyang University, Ansan, South Korea; 2 Yonsei University, Seoul, Korea

**Keywords:** Atmospheric dynamics, Physical oceanography

## Abstract

Characteristics of sea ice extent (SIE) have been rapidly changing in the Pacific Arctic sector (PAS) in recent years*.* The SIE variability in PAS during the late spring and early summer (i.e., April–May–June, AMJ) plays a key role in determining the SIE during the following fall when SIE is at a minimum. We find that the Pacific Decadal Oscillation (PDO), which is the most dominant variability of sea surface temperature (SST) on the low-frequency timescales, differently influences the SIE in PAS during AMJ before and after the mid-1990s. While a positive phase of PDO during the previous winter acts to increases SIE during AMJ before the mid-1990s, it acts to decrease SIE during AMJ after the mid-1990s. Further analysis indicates that atmospheric circulation associated with PDO differently influences the variability of SIE in the PAS during AMJ by modulating poleward moisture transport across the Alaska or the Far East Asia peninsula. This results in the change in the relationship of PDO and SIE in the PAS before and after the mid-1990s.

## Introduction

The record-breaking minimum year of the Arctic sea ice cover has been replaced every several years due to global warming^1–4^ and/or internal climate variability^3,5^. As a direct result of the Arctic sea ice loss, voyage across the Arctic Ocean is now possible, which has reduced the time required to cross the ocean and enabled the exploitation of new natural resources. Although it is a controversial issue, the reduction of sea ice cover could alter atmospheric circulation, resulting in severe extreme cold events and significant snowfall in North America and Eurasia during the boreal winter^6–8^. Therefore, predicting the variability of SIE in fall when SIE is at a minimum is important for economic benefits, human health, and safety. The variability of SIE in fall could be influenced by many relevant factors including atmospheric moisture, cloud, atmospheric heat transport, oceanic heat transport and sea ice motion^9–15^.

Combined factors may differently affect SIE in the Pacific Arctic sector (PAS) (140° E–130° W, 65°N–82°N) including Beaufort, Chukchi and East Siberian seas from year to year. However, it is well known that the SIE variability in late spring and early summer is a good indicator of SIE variability in September when SIE is at a minimum^16^. That is, preconditions for changes in sea ice in the following fall and early winter are associated with a sea ice loss in previous spring^17^. While ice-albedo feedback is not yet active until early spring since solar radiation merely reaches the surface, anomalous Arctic cloudiness or moisture convergences are primarily responsible for melting sea ice in early spring via increased downward longwave radiation^17,18^. On the other hand, there is a study argued that sea ice motion in previous winter is essential for sea ice thickness variability, which is also an indicator of the SIE in the following fall^14^. Therefore, it is crucial to understand the physical processes leading to the SIE variability in spring to predict the SIE in fall. In this study, we focus on the SIE variability during the late spring and early summer (April–May–June, hereafter, AMJ) by analyzing the reanalysis datasets. In particular, we first highlight the role of Pacific Decadal Oscillation (PDO), which is the most dominant variability of sea surface temperature (SST) in the North Pacific, on the variability of the SIE in the PAS during AMJ. While the PDO is dominant on the low-frequency timescales, it is also characterized by a considerable variability less than decadal timescales (Fig. S1). Such temporal characteristics are also observed in the variability of the SIE in the PAS (Fig. S2). Our analysis indicates that the role of the PDO during the late winter and early spring (January–February–March, hereafter, JFM) on the SIE variability in the following AMJ has changed from before and after the mid-1990s. A shift of PDO’s influence has not been examined in the previous literature.

We analyzed the change in SIE with respect to PDO index using monthly sea ice concentration (SIC) from National Oceanic and Atmospheric Administration/National Snow and Ice Data Center (NOAA/NSIDC)^19^. The importance of sea ice variability in AMJ is highlighted by displaying the seasonal auto lead-lag correlation of SIE. We then studied physical processes associated with the atmospheric circulation related to PDO, which affect SIE variability during late spring and early summer (AMJ) using reanalysis data (see “Methods” section).

## Results

**Figure 1:**
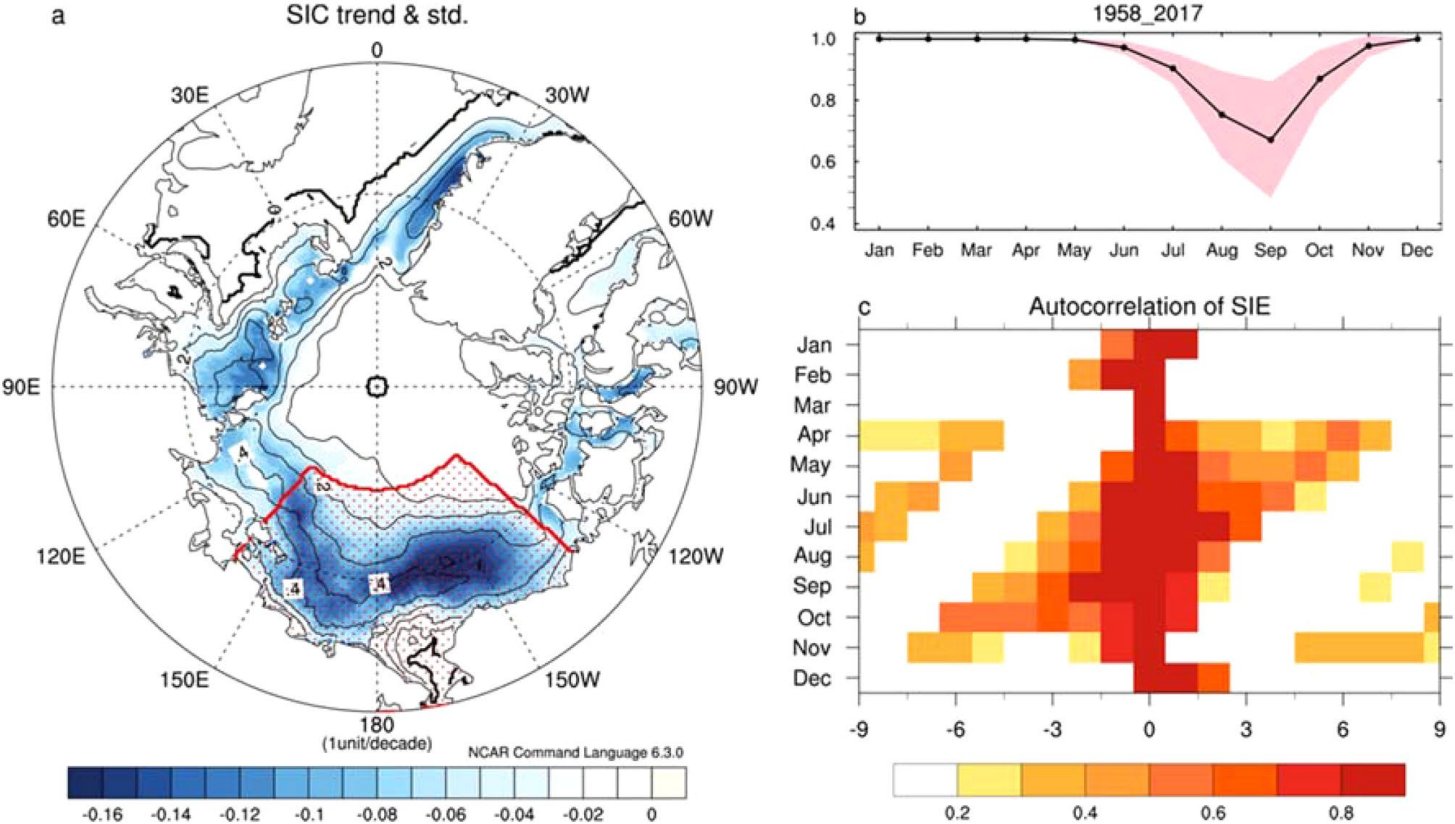
(**a**) Linear trend (shading) and standard deviation (contour) of sea ice concentration in September. The shaded area denotes the region where the statistical significance exceeds 90% of the confidence level according to Student’s t-test. The unit is 1 unit/decade. The contour interval is 0.1. (**b**) Seasonal cycle of SIE over the Pacific Arctic sector (PAS, 140° E–130° W, 65° N–82 °N, red dotted in (**a**). The pink shading indicates one standard deviation of SIE variability in the Pacific Arctic sector. (**c**) Seasonal cycle of autocorrelation of SIE in the PAS for 1958–2017. Y-axis denotes base month, and x-axis denotes lead-lag months. Before we calculate the autocorrelation, we calculate two months running mean with removing its trend. The correlation coefficients are displayed wherever confidence level exceeds 90% from the t-test. Note that the number of degree freedom is determined based on auto lag-1 correlation. Plots were generated using NCAR Command Language (https://doi.org/10.5065/D6WD3XH5) version of 6.3.0^44^.

Figure 1a displays the linear trend of SIC for the entire analyzed period (1958–2017) and its standard deviation in September. The greatest reduction of SIC in September since 1958 is observed in the PAS, marked by the red dotted area in Fig. 1a. In addition, the SIE variability is also large within the same area. Note that the area we take into account to define the PAS little affects to the main conclusion in the present study. Figure 1b shows the seasonal cycle of the SIE in the PAS, as well as its standard deviation for 1958–2017, indicating that the SIE variability is the largest in September when the SIE is at a minimum of a year. To further identify the characteristics of SIE variability in the PAS, we calculate the auto lead-lagged correlation of SIE anomaly in the PAS (Fig. 1c). While the auto-correlation coefficients of SIE in the PAS during JFM drop rapidly, that during AMJ is significantly positively correlated with that during the following season until early winter. Similarly, the SIE variability in the PAS during fall and early winter is significantly positively correlated with that during the previous season until late spring. That is, once the anomalous SIE is enhanced during AMJ, it is associated with an enhancement of the anomaly of SIE the following fall and early winter, and vice versa. A rapid decrease of the auto-correlation coefficients of SIE during JFM is because JFM is the nearest season when the SIE starts to melt. However, such persistency of SIE anomaly from AMJ to the following fall and early winter might be caused by a positive feedback process, including ice-albedo feedback^20,21^, which acts through the entire season. In particular, anomalous southerly wind also would alter the ice surface properties, thereby strengthening the ice-albedo feedback in the PAS^21^. In addition, previous studies also suggested the melt-to-growth sea ice reemergence mechanism, where spring sea ice anomalies are stored as upper ocean heat content anomalies and reemerge the following winter^15,22,23^. Simply put, Fig. 1c indicates that the variability of SIE during AMJ is closely associated with the amount of SIE in the PAS during the following fall and even early winter.

Among the possible factors that could affect the SIE variability in the PAS during AMJ, we consider the role of PDO during JFM. The climatological (1958–2017) seasonal cycle of radiative fluxes and oceanic heat fluxes and their standard deviations show that the oceanic heat transport and its variability is quite small across the Bering Strait compared to the radiative fluxes including shortwave, longwave, sensible and latent heat fluxes during JFM (Fig. S3). Therefore, we argue that the PDO and its associated atmospheric circulation may act to directly influence SIE variability compared to the role of oceanic heat transport (see also Table 1). Consistently, it is known that the variation of SIE during AMJ is in response to anomalous atmospheric radiative preconditions, i.e., downwelling longwave flux, with 2–3 lag months^24^. We also found that the downwelling long wave radiation during JFM is negatively correlated with the SIE during AMJ in the PAS during 1958–2017, i.e., − 0.43, which is statistically significant at the 95% confidence level. An anomalous downward longwave radiation acts persistently to reduce sea ice thickness, resulting in a thinning of sea ice in the same season^25^ (see also Fig. S4). Consequently, a thinner sea ice would easily reduce the extent in spring. A previous study also argued that enhanced poleward moisture transport from the North Pacific to the Arctic Ocean contributed to the accelerated SIE decrease during the most recent period when the thickness of sea ice became thinner^26–28^.

**Table 1:** Regressed heat flux anomalies in PAS during JFM and AMJ against with the PDO index in P1 (1958–1994) and P2 (1995–2017), respectively.

	P1(1958-1994)		P2(1995-2017)	
	JFM	AMJ	JFM	AMJ
Q_net_ (Wm^−2^)	− 0.55	− 0.21	0.95	− 0.16
Q_LW_ ↓ (Wm^−2^)	− 0.92	− 1.62*	3.16*	0.11
Q_LW_ (Wm^−2^)	− 0.48*	− 0.76*	0.25	− 0.19
Q_SW_ (Wm^−2^)	0.05*	0.21	− 0.07*	0.11
Q_SH_ + Q_LH_ (Wm^−2^)	− 0.13	0.34	0.77	− 0.08
vT65 (Wm^−2^)	0.01	0.17	0.03	0.20
WVP (10^−1^kgm^−2^)	− 0.08	− 0.96	1.41*	0.13

Net radiative heat flux, downwelling longwave radiation, net longwave radiation, net shortwave radiation, turbulent heat flux as a summation of sensible and latent heat flux, oceanic heat flux across 65° N and column integrated water vapor are denoted as Q_net_, Q_LW_↓, Q_LW_, Q_SW_, Q_SH_ + Q_LH_, vT65 and WVP, respectively. Flux anomalies are defined positive downward. Asterisk indicates a value exceeding 90% of the confidence level according to Student t-test. Oceanic heat flux across the Bering Strait approximately 65° N.

**Figure 2:**
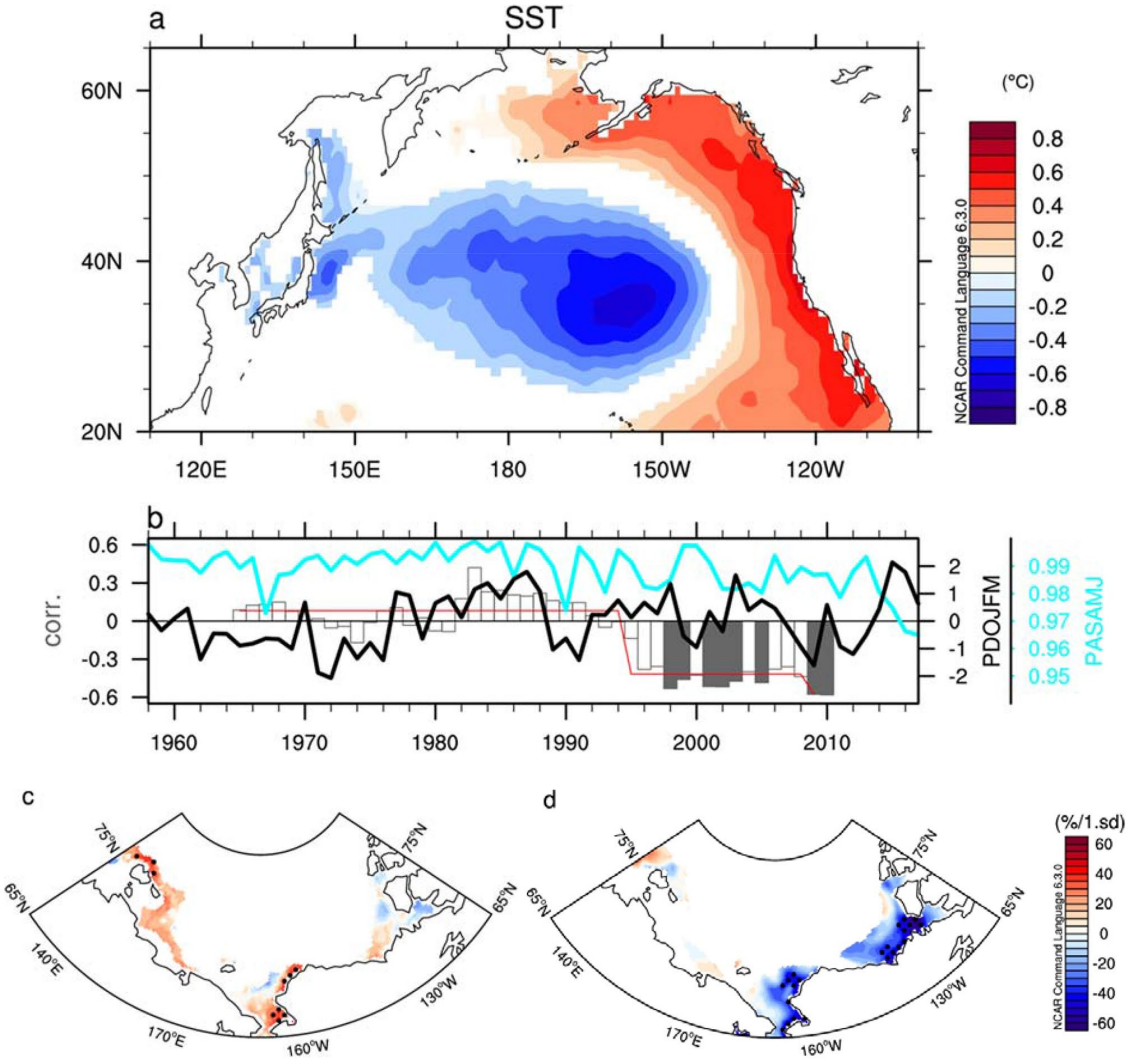
Shift of winter PDO’s influence on spring sea ice concentration (SIC). (**a**) Regressed map of SST in January, February, and March (JFM) averaged onto PDO index averaged in JFM for 1958–2017. Shaded color exceeds 90% of the significance level according to Student’s t-test. (**b**) Time series of the PDO JFM index is shown as a black solid line, and the cyan solid line indicates the SIC averaged over April, May, and June (AMJ) over PAS. Their correlation coefficient with a 15-year window is shown in the bar. Within each given window, the linear trend is removed in two variables. Gray bars indicate that the coefficient is statistically significant at the 90% confidence level from Student’s t-test. The red line indicates a regime shift analysis based on Rodinov’s sequential t-test^31^. The detection is performed with significance level of 0.1 and cut-off length of 15 years and Huber’s weight parameter 1.5. (**c**) Regression maps of SIC onto PDO JFM index during 1958–1994. (**d**) As in Fig. 2c but for 1995–2017. Plots were generated using NCAR Command Language (https://doi.org/10.5065/D6WD3XH5) version of 6.3.0^44^.

We display the spatial manifestations of the positive phase of PDO during JFM, which are characterized by cool temperatures in the western and central North Pacific with an elliptical shape and are accompanied by anomalously warm temperatures to the east, north, and south, and the opposite is true in the negative PDO phase (see Fig. 2a). Therefore, the variability of SIE in the PAS could be easily influenced by the PDO, because it is closely associated with the SST condition around the PAS (Figs. 1a, 2a). In spite of adjacent geographical conditions, however, correlation coefficients between the PDO index during JFM and the SIE variability during AMJ with and without the linear trend are − 0.14 and − 0.02 for 1958–2017, which are negligible. Because of the low correlation, there are few studies evaluating the role of the PDO on the SIE variability in the PAS. However, we argue that this negligible correlation is mainly due to a dramatic change in the relationship of PDO and SIE variability.

We found that the relationship of JFM PDO and AMJ SIE with a 15-year running window has been significantly changed across the mid-1990s based on Rodionov’s sequential t-test analysis^29^ (Fig. 2b). While the PDO during JFM is positively correlated with the variability of SIE in AMJ before the mid-1990s, a negative relationship of JFM PDO and AMJ SIE is significant at the modest level of 90% confidence after the mid-1990s. That is, a positive phase of PDO acts to decrease the SIE during AMJ prior to the mid-1990s and vice versa subsequently (see also Fig. 2c,d). Note that a running correlation coefficient is calculated after we removed a linear trend in every 15-year window. It is also noteworthy that the correlation coefficient between JFM PDO and AMJ SIE is 0.29 during 1958–1994 and −0.58 during 1995–2017, respectively, which is statistically significant at the 90% confidence level. Despite the correlation does not imply the causality of PDO’s influence on SIE variability in the PAS, we hypothesize that the influence of atmospheric condition associated with the PDO on the SIE variability in the PAS has dramatically changed since the mid-1990s.

**Figure 3:**
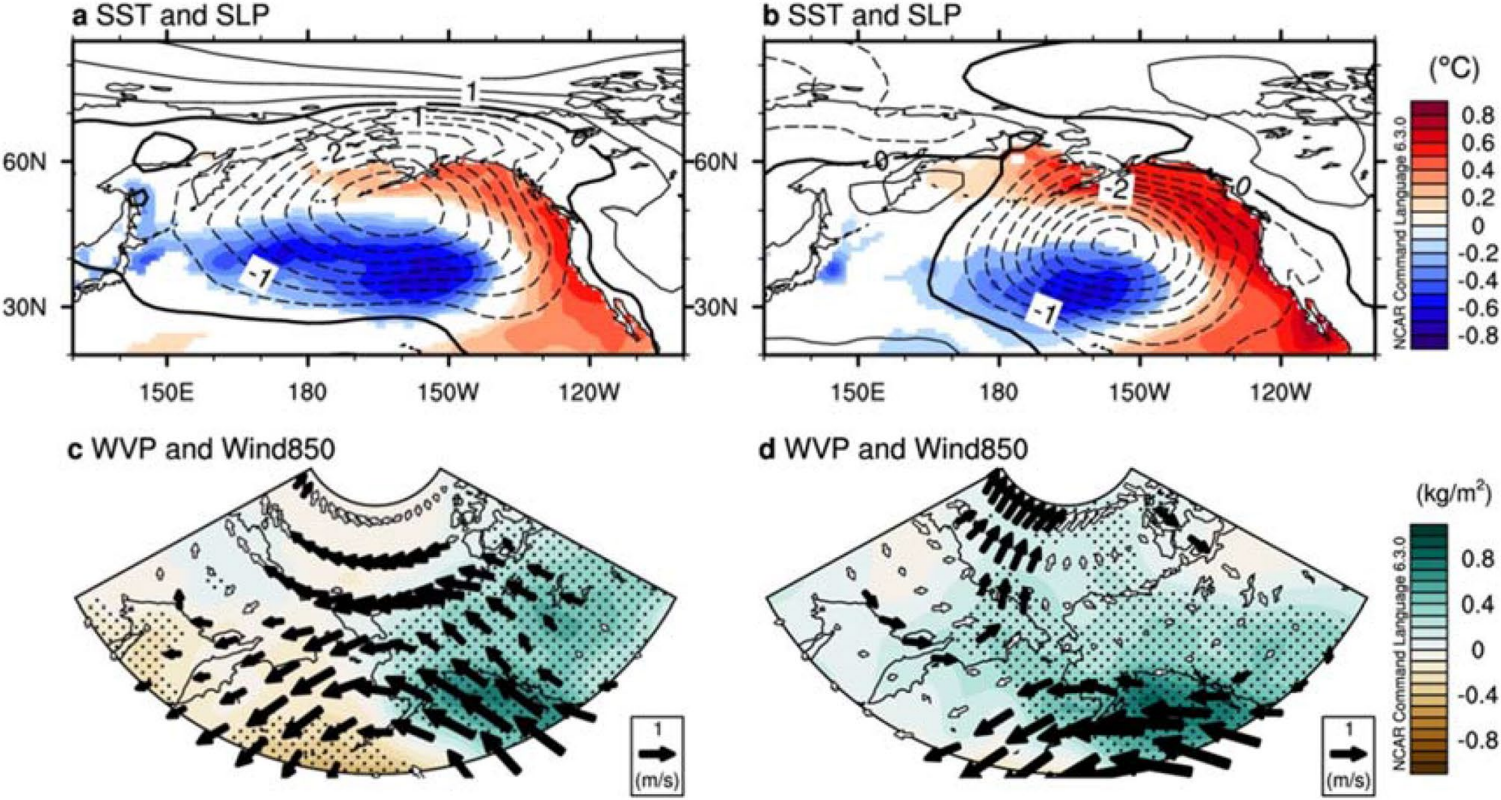
Regression maps of surface sea temperature (SST) and sea level pressure (SLP) in JFM onto PDO JFM in P1(1958–1994) (**a**) and P2 (1995–2017) (**b**). Shadings indicate SST in °C, and contour indicates SLP in hPa. Contour intervals are 0.5 hPa. SST exceeding 90% of the confidence level according to Student’s t-test is displayed. Regression maps of column integrated water vapor (WVP) and wind vector at 850 hPa, where water vapor is concentrated of levels, in AMJ as to each PDO JFM in P1 (**c**) and P2 (**d**). Dotted area indicates the region where anomalous WVP exceeds 90% of the confidence level according to Student’s t-test. Arrows filled with black indicate either of anomalous zonal or meridional winds exceeding 90% confidence level according to Student’s t-test. Units are m/s for the wind vectors and kg/m^2^ for WVP. Plots were generated using NCAR Command Language (https://doi.org/10.5065/D6WD3XH5) version of 6.3.0^44^.

To examine the details of the PDO’s role, we compared the two periods, before (1958–1994, hereafter, P1) and after the mid-1990s (1995–2017, hereafter, P2), respectively. Figure 3a,b displays the regressed SST and Sea Level Pressure (SLP) with respect to the PDO index during JFM at P1 and P2, respectively. The spatial pattern of the regressed SST anomalies associated with a positive PDO phase shows similarities and differences between the two periods. In particular, a center of a cool SST is shifted to the south in P2, and the warm SST around the Bering Sea and the west coast of North America is prominent in P2 compared to that in P1.

According to previous studies^30,31^, the mid-latitude North Pacific SST variability including PDO is able to modify the intensity and location of oceanic fronts, subsequently, it leads to modify the atmospheric circulation. We calculated the meridional SST gradient associated with the PDO, which is associated with the characteristics of baroclinic instability in the North Pacific, between the two periods (Fig. S5) and found that their spatial structures differ. Consistently, the zonal (150° E–140° W) mean zonal wind structure associated with PDO has been changed in two periods (Fig. S6). This could affect the meridional circulation in the North Pacific, which is associated with the change in the structure of moisture transport. It is also found that a center of the Aleutian Low (AL) associated with the PDO in JFM is shifted to the southeast in P2 compared to that in P1 (Fig. 3a,b). In addition, a center of high pressure in the PAS is shifted to northwestern Canada from P1 to P2, leading to a dipole-like structure of atmospheric circulation in the meridional direction in the northern part of North Pacific in P2 (Fig. 3b and see also Fig. S6b). These results indicate that the North Pacific SST anomalies associated with the PDO in JFM and its associated atmospheric circulations have been modified in P2. Therefore, the changes in atmospheric circulation associated with the PDO in JFM modify the structure of moisture transport into the PAS in AMJ between P1 and P2. Anomalous poleward moisture transport associated with a positive PDO phase during JFM is observed in P2 during AMJ (Fig. 3d), which is in contrast to that in P1 (Fig. 3c).

**Figure 4:**
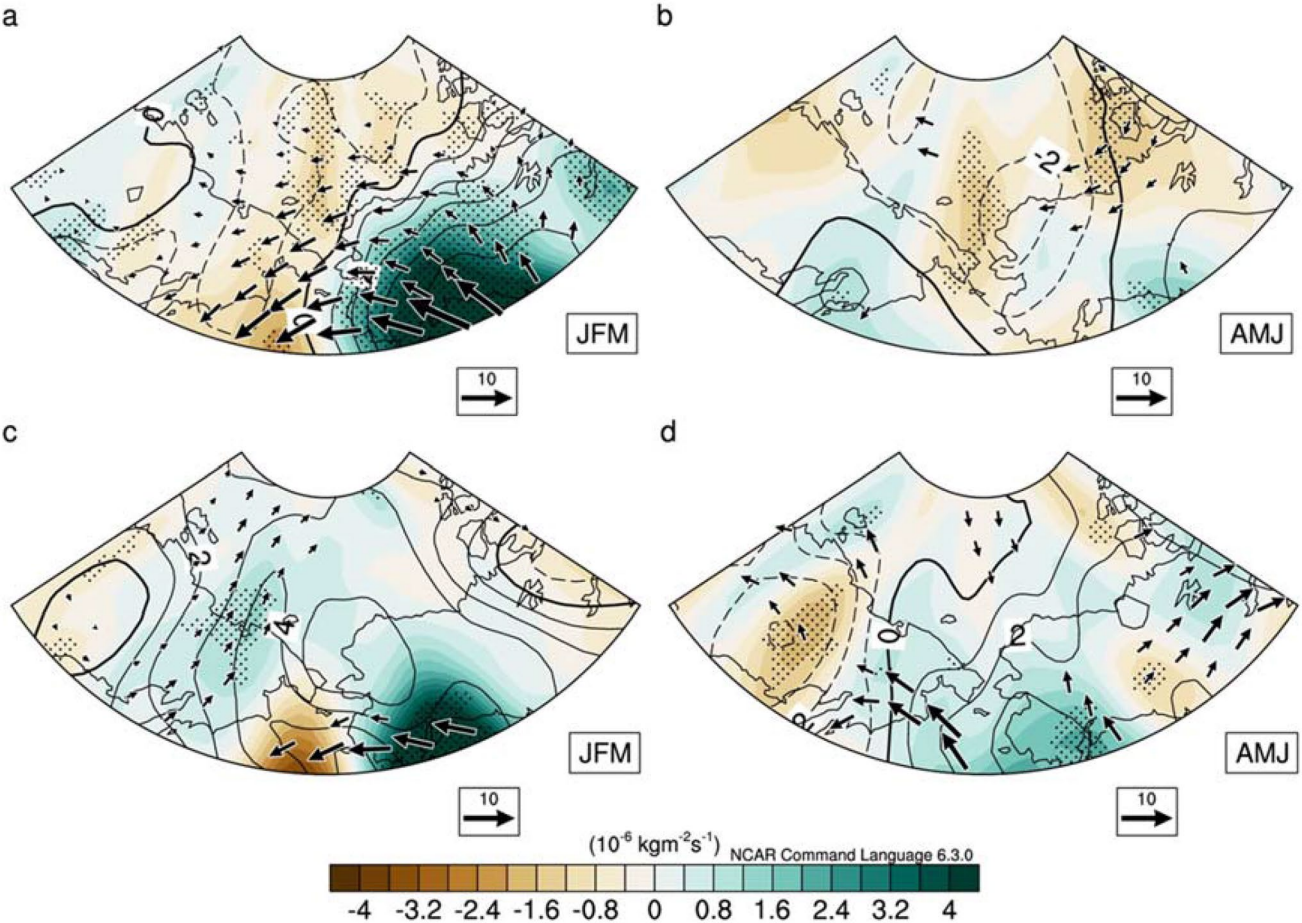
Regression maps of vertical integral of moisture flux in units of kg m^-1^ s^-1^ and their convergence (shading) in units of 10^–6^ kg m^-2^ s^-1^, downward long wave radiation (contours) in W m^-2^ at the surface in (**a**) JFM and (**b**) AMJ, respectively, onto PDO JFM in P1 (1958–1994). (**c**, **d**) The same as in (**a**, **b**) except but in P2 (1995–2017). Dotted area indicates the region where anomalous convergence of moisture fluxes exceeds 90% of the confidence level according to Student’s t-test. Vectors either of zonal or meridional fluxes above 90% of the confidence level are displayed. Plots were generated using NCAR Command Language (https://doi.org/10.5065/D6WD3XH5) version of 6.3.0^44^.

Figure 4a,b displays the regressed column integrated moisture fluxes, their convergence and downward longwave radiation during JFM and AMJ against the PDO index during JFM, respectively, in P1. Figure 4c,d are identical to Fig. 4a,b except that in P2. It is noted that the amount of integrated water vapor is large in the regions where downwelling longwave radiation is also anomalously large. This indicates that the amount of moisture, which is a strong greenhouse gas, is different in the PAS between P1 and P2 during a positive PDO phase and vice versa. During a positive PDO phase in P1, the amount of moisture decreased in the PAS, which results in an increased SIE through the reduction of downwelling longwave radiation (Figs. 3c, 4a,b). In contrast, the amount of moisture increased in the PAS during a positive PDO phase in P2, which results in a decreased SIE through the enhancement of downwelling longwave radiation (Figs. 3d, 4c,d). This results in a change in the PDO and SIE relationship between the two periods, which is attributed to the change in the role of atmospheric circulation associated with the PDO from P1 to P2. To understand the role of atmospheric warming trends and their associated increased poleward moisture transport, we also calculate the moisture fluxes explained by anomalous winds and climatological specific humidity (Fig. S7). We find that the spatial patterns of moisture fluxes, their convergence and downward longwave radiation are not much changed compared with the results in Fig. 4. This may indicate that the anomalous wind associated with the atmospheric circulation in each period plays a key role to change the relationship of PDO and SIE across the mid-1990s.

We also conduct heat budget analysis to confirm the notion that the anomalous downwelling longwave radiation associated with the PDO during JFM is dominant among other atmospheric radiations, resulting from the anomalous moisture transport (Table 1). While the oceanic heat transport through the Bering Strait is an important component to determine SIC anomalies in the PAS^12^, we find that the anomalous ocean heat transport associated with PDO is smaller than the anomalous downward LW and its role on the relationship of PDO and SIE is negligible (Table 1). The results in Table 1 indicate that the anomalous integrated water vapor and its associated downward longwave radiation plays a role to change the role of JFM PDO on the AMJ SIE in the PAS after the mid-1990s. Note that the net longwave radiation (LW) in JFM with respect to PDO JFM in P2 is relatively smaller than downwelling longwave radiation and it is possibly offset by anomalous upwelling longwave radiation due to high surface temperature through sea ice thinning.

**Figure 5:**
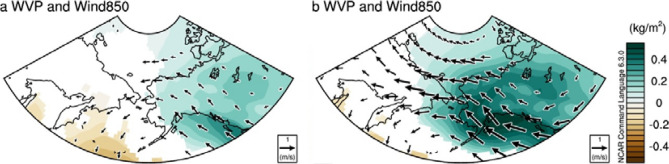
(**a**, **b**) The same as in Fig. 3c, d except but for GOGA simulation. After regressing WVP, winds at 850 hPa onto PDO JFM in each ensemble, their ensemble averages are calculated in P1(1958–1994) and P2(1995–2015), respectively. The shaded is displayed only when the anomalies in at least 8 ensembles agree about the sign. Due to the lack of data availability since 2016, the P2 in GOGA is limited to the period of 1995–2015. Plots were generated using NCAR Command Language (https://doi.org/10.5065/D6WD3XH5) version of 6.3.0^44^.

Further investigation has been performed using atmosphere-only climate model experiments, i.e. Global Ocean Global Atmosphere (GOGA) experiments (see “Methods” section). In spite of some discrepancies, we find that there are distinct spatial structures of winds and column integrated water vapor associated with PDO JFM between P1 and P2 (Fig. 5a,b). It is evident that the atmospheric circulations associated with the PDO in JFM have been modified from P1 to P2. Subsequently, this leads to an increase in the amount of column integrated water vapor in PAS during P2 compared to that during P1 by altering the structure of moisture transport into the PAS in AMJ between P1 and P2. This result supports the notion that the atmospheric circulation associated with SST (i.e., PDO) is able to modulate poleward moisture transport into the PAS, which may cause to change its associated SIE variability.

## Discussion

In this study, we focused on the variability of SIE in the PAS during AMJ due to its significant correlation with the following fall and winter. In particular, we examined the role of the PDO during JFM on the variability of SIE during AMJ. We found that the spatial pattern of PDO during JFM, which is associated with the structural changes in the meridional SST gradient as well as the zonal mean zonal wind in the North Pacific, differs significantly before and after the mid-1990s. This could affect the atmospheric circulation associated with the PDO across the mid-1990s, resulting in the changes in the relationship of PDO during JFM and SIE in the PAS during AMJ via modifying the poleward moisture transport in P1 and P2, respectively.

While we emphasized the role of PDO associated with atmospheric thermodynamic processes, we can not exclude that the processes associated with the sea ice thickness which depends on the inflow of multi-year ice with their sea ice-albedo feedback play a role for the loss of sea ice in the PAS^21,32^. We found that the characteristics of anomalous sea ice thickness with sea ice motion associated with the PDO change across the mid-1990s (Fig. S8). The anomalous sea ice motion associated with PDO acts to redistribute the anomalous sea ice thickness in JFM. While the sea ice becomes thick in the south of East Siberian Sea with the anomalous southward ice transport in a positive phase of PDO in P1, that associated with PDO is not significant in P2. Therefore, the change in the relationship of PDO-SIE in the PAS could be also associated with the change in sea ice thickness and its associated sea ice motion associated with PDO.

While the PDO during JFM is not a good predictor for the SIE during the following fall and winter (i.e., October–November–December, OND) for the entire analyzed period of 1958–2017 (r = 0.04, here r is a correlation coefficient between JFM PDO and OND SIE), the correlation coefficient between JFM PDO and OND SIE is 0.35 in P1 and − 0.35 in P2, respectively, which is statistically significant at the 90% confidence level. Therefore, the PDO index could be used to predict the SIE during OND in different periods, which is consistent with the results in the present study. Although the linear trend does not imply the radiative forcing, on the other hand, the overall results with and without a linear trend little change. Therefore, we speculate that the role of radiatively-forced trend on the impact of the PDO on the SIE variability is not significant.

## Methods

### Data

The PDO index, which is defined as the principal component time series of the first empirical orthogonal function SST anomalies in the North Pacific poleward of 20° N^33^, is obtained from the University of Washington (https://jisao.washington.edu/pdo/PDO.latest). In this study, we mainly use the PDO index averaged during JFM, when the PDO intensity is maximized^34^. Monthly SST is obtained from Hadley Centre SST (HadISST1) data set with resolution of 1° × 1°^35,36^. Monthly sea ice concentration (SIC) at a spatial resolution of 25 km is obtained from National Oceanic and Atmospheric Administration/National Snow and Ice Data Center (NOAA/NSIDC) for the period of 1979–2017^19^. We also used gridded SIC with 0.25° × 0.25°  from^37^ prior to 1979 and reconstructed the SIC dataset having a spatial resolution of 25 km. Note that the results little change when we used the SIC from the ERA-Interim reanalysis dataset. The entire analyzed period is 1958–2017 and the analysis period starts in 1958 because of data credibility issue^38^. It is noteworthy that the results in the present study are little changed when the analyzed period is limited after 1979.

Monthly sea ice thickness and ice motion vectors are obtained from Pan-Arctic Ice Ocean Modeling and Assimilation System (PIOMAS)^39^ available from 1979. The data is gridded to 25 km × 25 km. Monthly precipitable water content; sea level pressure; surface pressure; specific humidity, zonal and meridional winds at 8 pressure levels from 1000 to 300 hPa with horizontal resolution of 2.5° × 2.5° are obtained from Reanalysis 1 of the National Centers for Environmental Prediction-National Center for Atmospheric Research (NCEP-NCAR)^40^. We also analyzed heat fluxes including shortwave radiation, longwave radiation, latent heat flux and sensible heat flux at surface from NCEP-NCAR Reanalysis 1 with horizontal resolution of T62 (192 × 94). The total heat flux at surface is obtained from the summation of four heat fluxes components as follows,

$${\text{Q }} = {\text{ Q}}_{{{\text{SW}}}} + {\text{ Q}}_{{{\text{LW}}}} + {\text{ Q}}_{{{\text{SH}}}} + {\text{ Q}}_{{{\text{LH}}}} ,$$

where Q_SW,_ Q_LW,_ Q_SH,_ and Q_LH_ indicate the net shortwave flux, net longwave flux, sensible heat flux, and latent heat flux, respectively. Q_SW,_ and Q_LW_ are composed of downwelling and upwelling components, respectively. From the viewpoint of the surface, sign of flux is positive when it downwells so that it warms the surface.

### Vertically integrated moisture flux

Convergence of vertically integrated moisture flux is calculated using specific humidity, zonal and meridional winds at 8 levels from surface pressure to 300 hPa. The vertically integrated moisture flux can be written as,

$$\vec{Q} = \frac{1}{g}\mathop \smallint \limits_{Ps}^{{300{\text{hPa}}}} q\vec{V}dp$$

where q is specific humidity, $\vec{V}$ is horizontal wind vector, p is pressure,${ }P_{S}$ is surface pressure and g is the gravitational acceleration. Since specific humidity above 300 hPa is negligible^41^, the vertical integration is performed from surface to 300 hPa. The SIE is defined as the sum of the area of gridcell wherever SIC is at least 15% in PAS (140–230 °E, 65–82 °N). When sea ice is completely covered, the SIE in the PAS is approximately 3.26 million km^2^, which is equivalent to 1 unit of SIE used in this study. The climatological period is defined as 1958–2017 to obtain the SIE anomaly.

### Oceanic heat transport across the Bering Strait

Ocean potential temperature and meridional currents with 1° × 1° horizontal resolution at 42 levels are from European Center for Medium-Range Forecasting’s Ocean Reanalysis System 4 (ORAS4)^42^. Oceanic heat flux across the Bering Strait is calculated from the ORAS4 data using the following equation:

$$\mathop \smallint \limits_{167^\circ W}^{^\circ W} \mathop \smallint \limits_{ - 50m}^{0} \rho C_{w} \left( {\theta - \theta_{ref} } \right)vdzdx$$

where ρ is the density of sea water (1023 kg m^-3^), *C*_*w*_ is the specific heat capacity of water (3900 J kg^-1^ K^-1^).

### Regime shift analysis

To determine the period when the relationship between PDO and SIE is different, we employed the regime shift analysis using Rodinov’s sequential t-test^29^. Based on this methodology, the periods before and after the mid-1990s (1958–1994 and 1995–2017) are considered separately to examine the change in the relationship between PDO during JFM and SIE variability in the PAS during AMJ. Note that the least squares linear trends of the analyzed variables are removed in each period.

### GOGA experiment

Global Ocean Global Atmosphere (GOGA) simulation using the Community Earth System Model version 1 (CESM) is used for the analyzed period of 1958–2015. Total 10 ensemble members are integrated from the same initial condition with a small change in 2 m temperature. The historical SST and sea ice, which is used to force the CESM, is obtained from Extended Reconstructed Sea Surface Temperature version 4 (ERSSTv4)^43^ and HadISST1, respectively.

### Data availability

The data that support the findings of this study are openly available. The SIC data used in this study is from NOAA/NSIDC available at https://nsidc.org/data/seaice_index and the SST data is available from the UK Meteorological Office Hadley Centre at https://www.metoffice.gov.uk/hadobs/. The SIT and ice motion vectors are available from from the PIOMAS at https://psc.apl.uw.edu/research/projects/arctic-sea-ice-volume-anomaly/data/model_grid. NCEP-NCAR reanalysis data is available at https://www.esrl.noaa.gov/psd/. Finally, ORAS4 ocean potential temperature and meridional currents are available at https://www.ecmwf.int/en/research/climatereanalysis/ocean-reanalys. The GOGA simulation data is downloaded via CESM website.

## Supplementary information

Supplementary Information 1.
