# Supplementary material for: Changes in the role of Pacific decadal oscillation on sea ice extent variability across the mid-1990s
Source: Sci Rep. 2020 Oct 16;10:17564. doi: 10.1038/s41598-020-74260-0 (PMC7567812; doi:10.1038/s41598-020-74260-0)
Supplement: Supplementary file 1 — Supplementary Information 1. [file 41598_2020_74260_MOESM1_ESM.docx]

**Supplementary information**

**Changes in the role of Pacific Decadal Oscillation**

**on sea ice extent variability across the mid-1990s**

Hyerim Kim,*^1^* Sang-Wook Yeh,*^1^* Soon-IL An,*^2^* and Se-Young Song,*^1^*

*^1^Hanyang University, ERICA, Ansan, Korea*

*^2^Yonsei University, Seoul, Korea*

*Submitted to Scientific Reports*

*June 2020*

*Corresponding Authors: Sang-Wook Yeh (*[*swyeh@hanyang.ac.kr*](mailto:swyeh@hanyang.ac.kr)*), Hanyang University, Ansan, South Korea, Soon-Il An (sian@yonsei.ac.kr), Yonsei University, Seoul, South Korea*


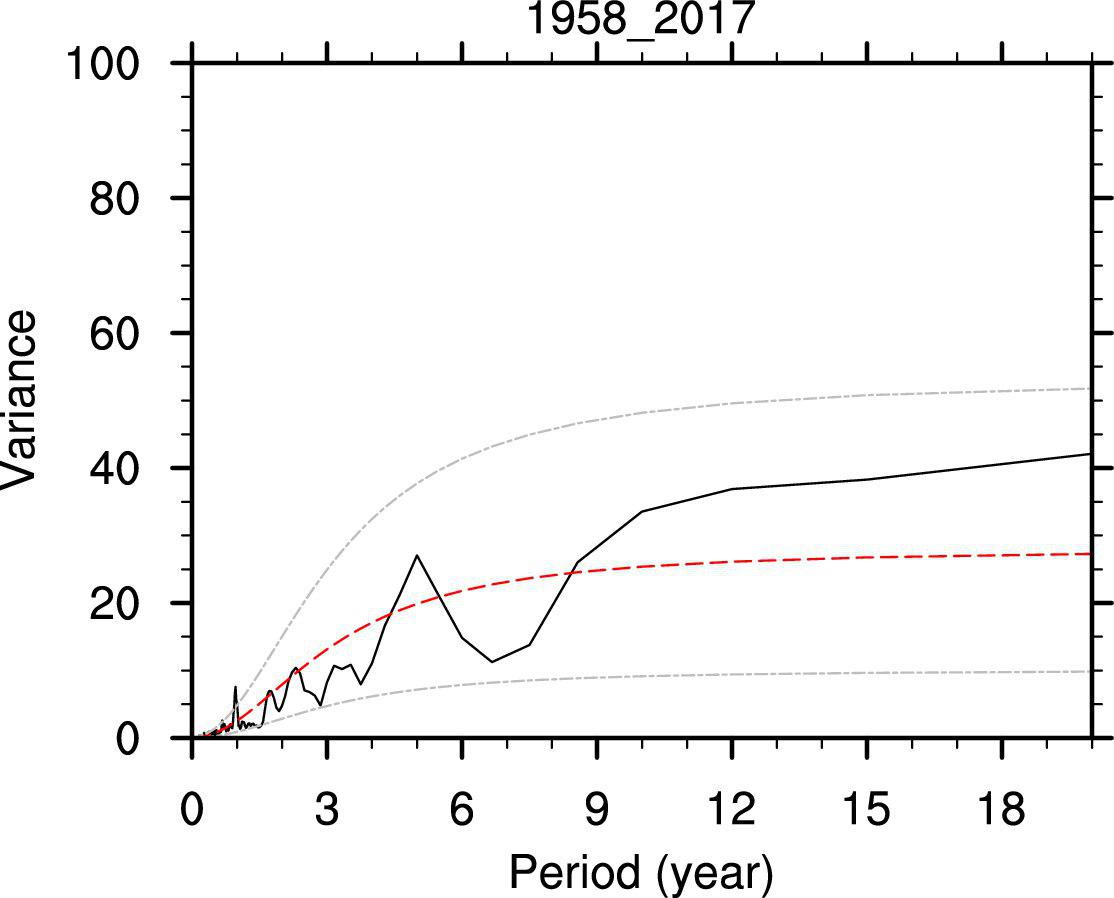


Figure S1 Spectral power of monthly PDO index from JISAO during 1958-2017. The least squares linear trend is removed. Red lines indicate red noise levels. The upper and lower gray lines indicate 95% and 5 % significance levels. Plot was generated using NCAR Command Language (<https://doi.org/10.5065/D6WD3XH5>) version of 6.3.0^44^.


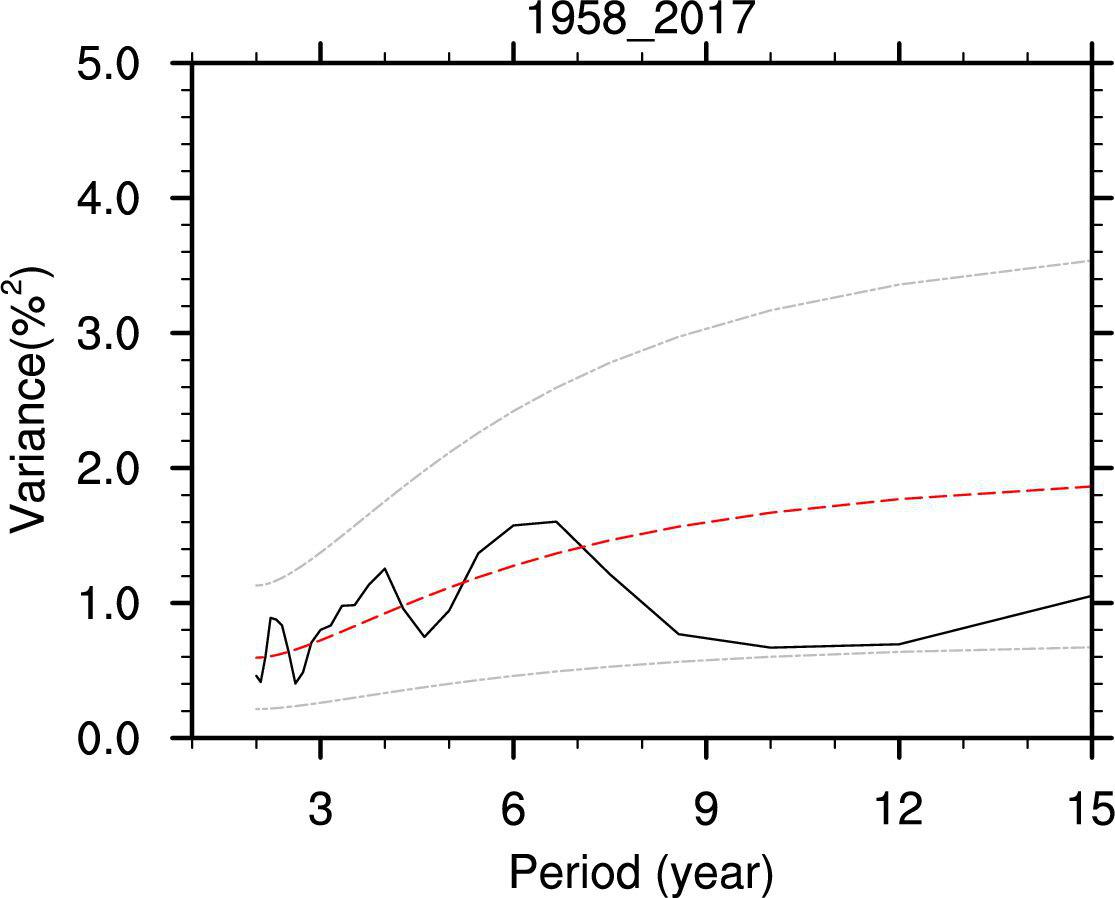


Figure S2 Spectral power of sea ice extent(SIE) in AMJ from NSIDC during 1958-2017. The least squares linear trend is removed. Red lines indicate red noise levels. The upper and lower gray lines indicate 95% and 5 % significance levels. Plot was generated using NCAR Command Language (<https://doi.org/10.5065/D6WD3XH5>) version of 6.3.0^44^.


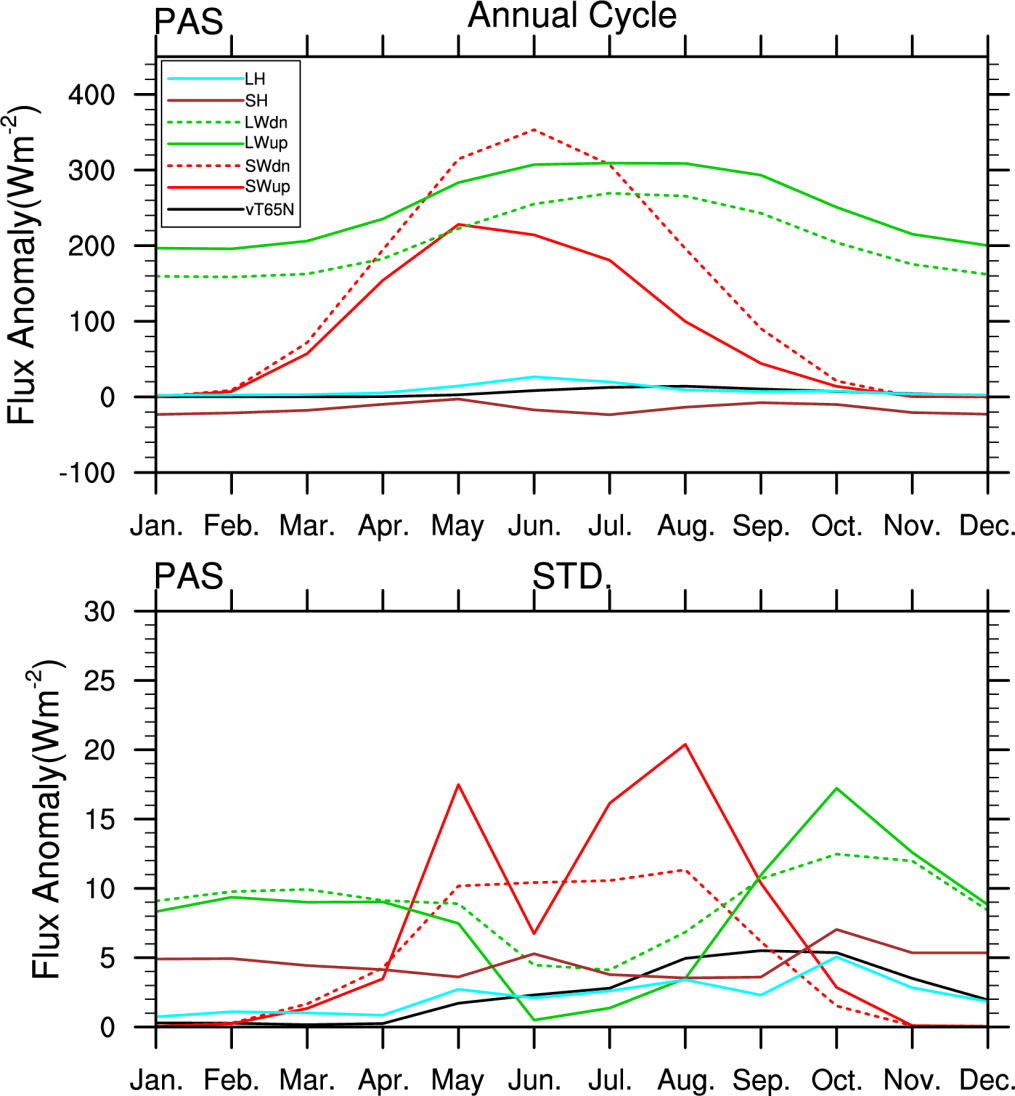


Figure S3 Seasonal cycle of radiative fluxes (upper) and its standard deviation (bottom) for the period of 1958-2017. Downward (dn, dashed) and upward (up, solid) shortwave (SW, red) and longwave (LW, green) radiations, sensible heat flux (SH, brown), latent heat flux (LH, cyan) and oceanic heat transport across the Bering Strait (vT65N, black). Plots were generated using NCAR Command Language (<https://doi.org/10.5065/D6WD3XH5>) version of 6.3.0^44^.


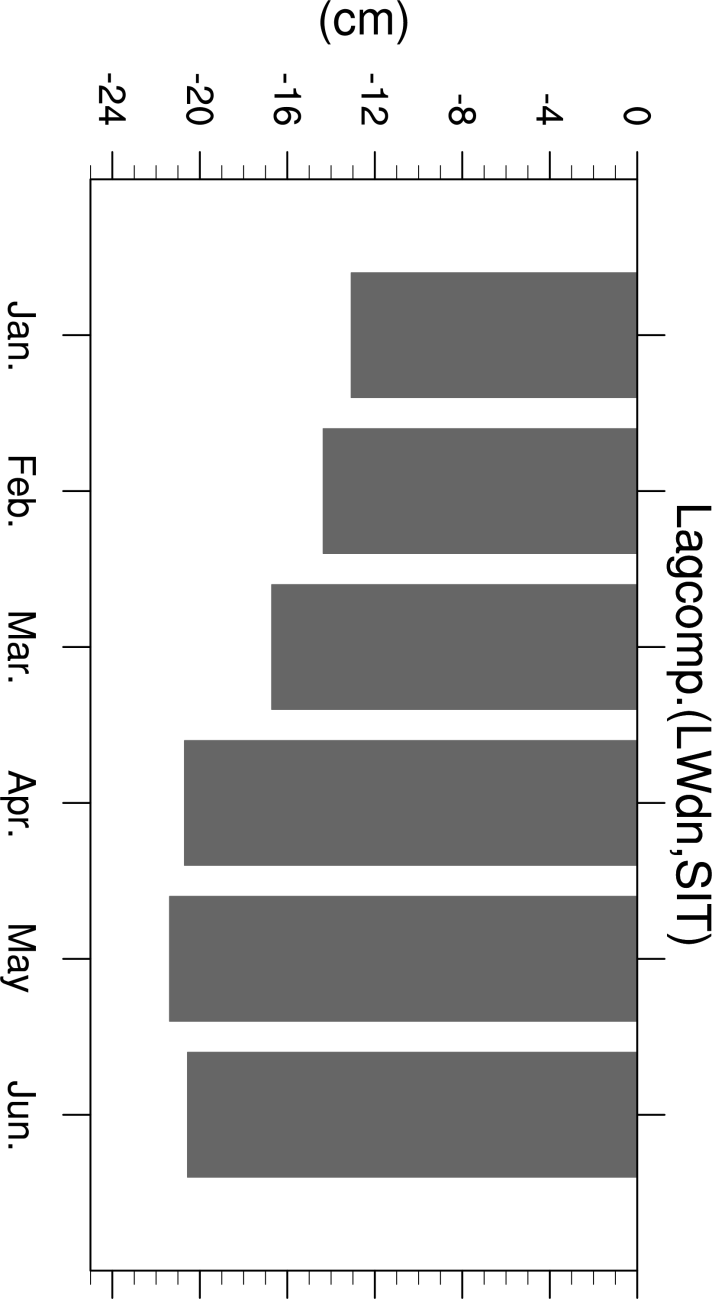


Figure S4 Composite of sea ice thickness (SIT) anomalies from January to June when the downward longwave radiation averaged in the PAS during JFM is above one standard deviation for the period of 1979-2017. Note that the composite years include 1981, 1989, 1996, 2005, 2011, 2014 and 2017 and the linear trends in all variables including the downward longwave radiation and the SIT are removed. The anomalies in all the months are statistically significant at 95% confidence level. The SIT for the period of 1979-2017 is from Pan-Arctic Ice Ocean Modeling and Assimilation System (Zhang and Rothrock, 2003). Plots were generated using NCAR Command Language (<https://doi.org/10.5065/D6WD3XH5>) version of 6.3.0^44^.


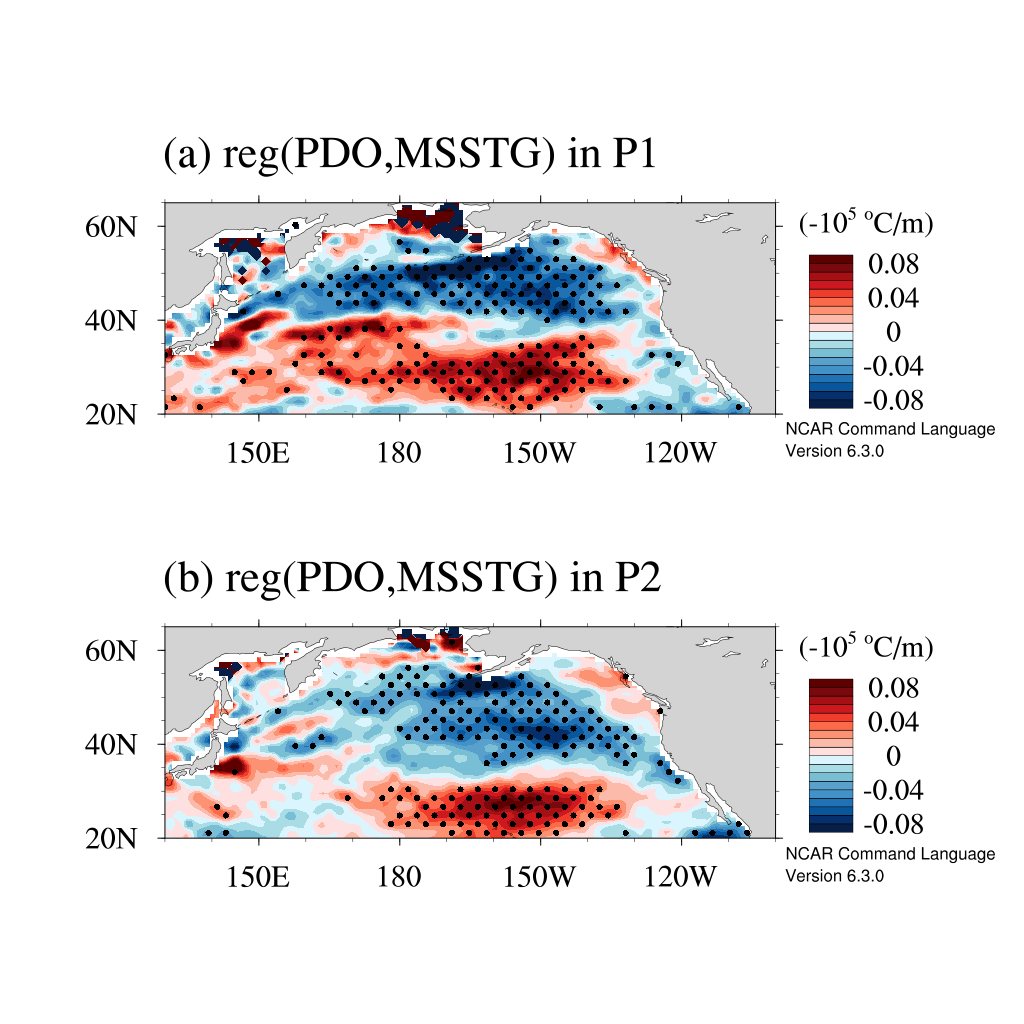


Figure S5 Regression maps of meridional sea surface temperature gradient in JFM onto PDO JFM in P1 (1958-1994) (a) and P1 (1995-2017) (b). Dotted area indicates the region where the meridional sea surface temperature gradient exceeds 95 % of the confidence level according to Student’s t-test. Units are °C/m. Plots were generated using NCAR Command Language (<https://doi.org/10.5065/D6WD3XH5>) version of 6.3.0**^44^**.


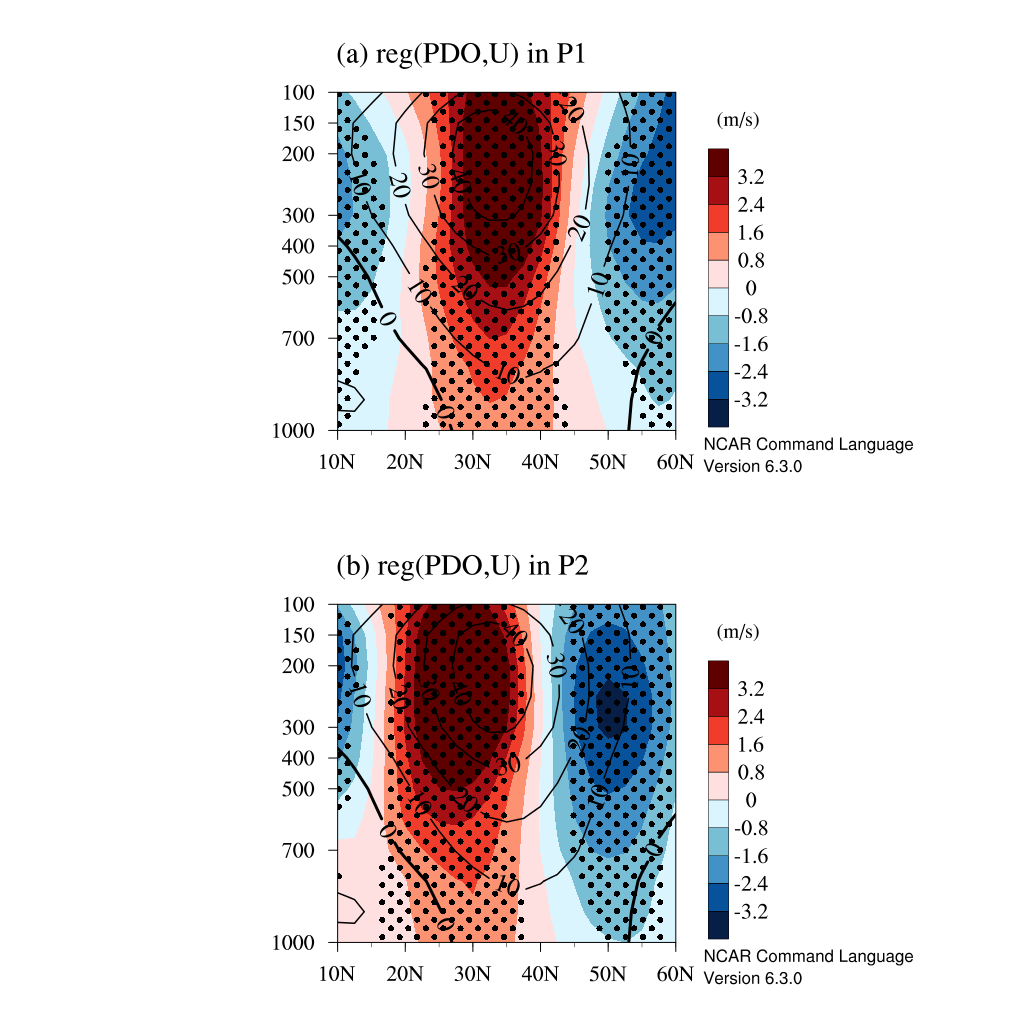


Figure S6 Vertical section of regressed zonal wind anomaly (shading) and in JFM onto PDO JFM in P1 (1958-1994) (a) and P1 (1995-2017) (b). Contours indicate climatological zonal wind during 1958-2017. Zonal band covers from 150°E to 140°W. Dotted area indicates the region where anomalous MSSTG exceeds 95 % of the confidence level according to Student’s t-test. Units are m/s. Plots were generated using NCAR Command Language (<https://doi.org/10.5065/D6WD3XH5>) version of 6.3.0^44^.


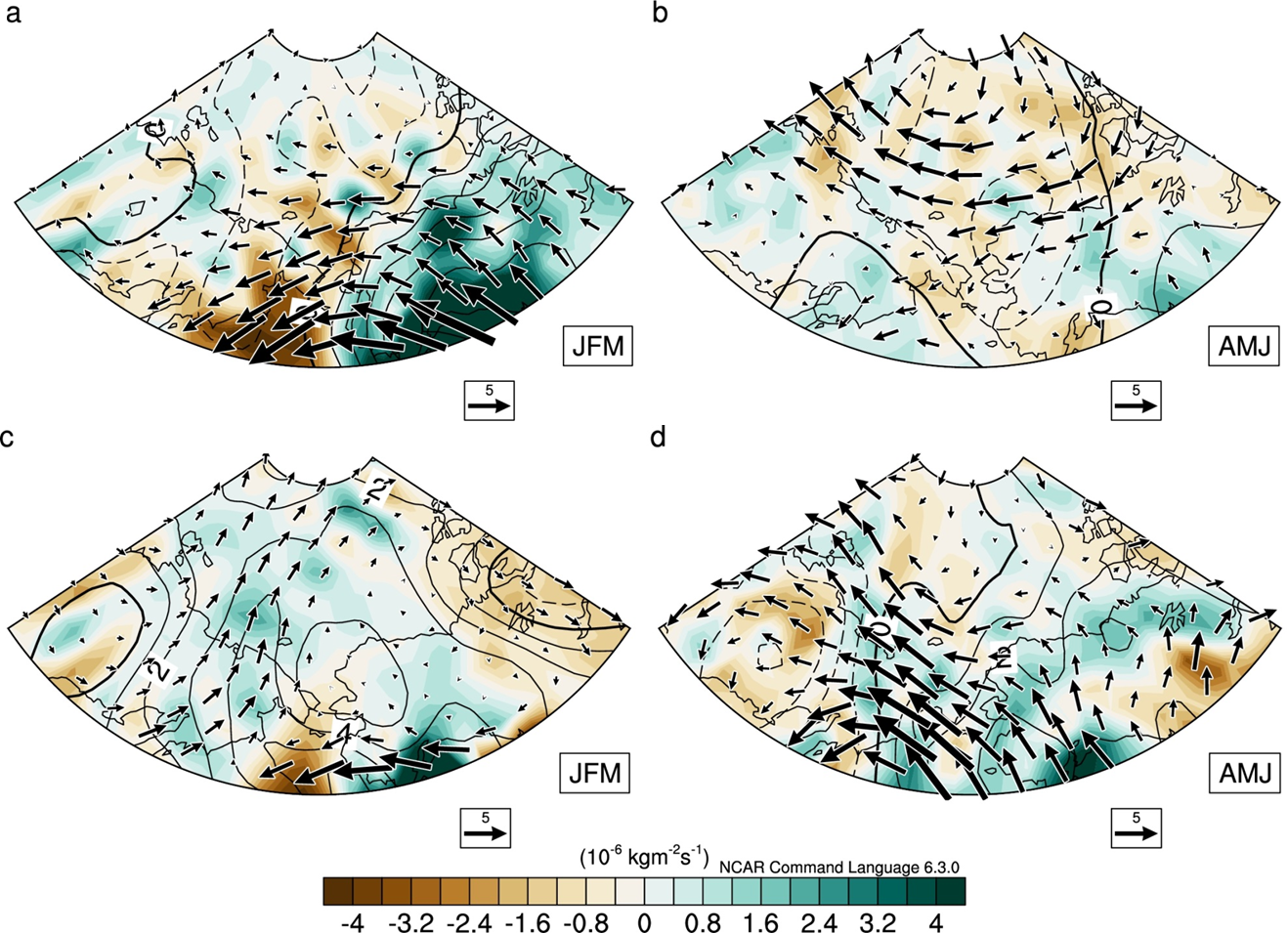


Figure S7. The same as in Fig. 4 except the moisture fluxes explained by anomalous winds and climatological (1958-2017) specific humidity. Plots were generated using NCAR Command Language (<https://doi.org/10.5065/D6WD3XH5>) version of 6.3.0^44^.


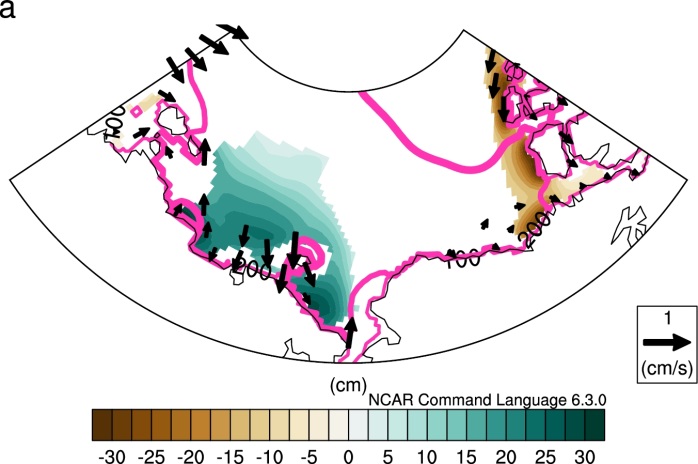

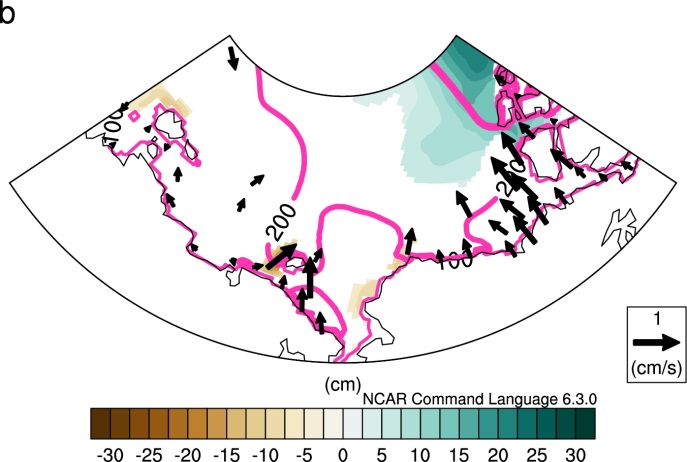


Figure S8. Regression maps of sea ice thickness (SIT) and sea ice motion vector in JFM onto PDO JFM in P1`(1979-1994) (a) and P2 (1995-2017) (b). Shadings indicate anomalous SIT and pink contours indicate respective climatological SIT. The regressed SIT exceeding 90% of the confidence level according to Student’s t-test is displayed. Unit in SIT and sea ice motion is cm and cm/s, respectively. Plots were generated using NCAR Command Language (<https://doi.org/10.5065/D6WD3XH5>) version of 6.3.0^44^.
